# Gomez Technique Combined With Modified Hosseini Technique: Two Underrecognized Techniques Toward a Potential Gold Standard for Posterior Anastomotic Urethroplasty

**DOI:** 10.7759/cureus.102023

**Published:** 2026-01-21

**Authors:** Jihad El Anzaoui, Ali Akjay, Najwa Jmil, Nawfal Ettoumi, Sohaib Bidan, Pankaj Joshi

**Affiliations:** 1 Urology, University Sidi Mohamed Ben Abdellah, Meknes, MAR; 2 Urology, Moulay Ismail Military Hospital, Meknes, MAR; 3 Urology, Kulkarni Center for Reconstructive Urology, Pune, IND

**Keywords:** bulbar arteries sparing urethroplasty, pelvic fracture urethral injury, urethral necrosis, urethral stricture, urethroplasty

## Abstract

Sparing of the bulbar arteries during posterior post-traumatic urethroplasty remains a relatively underrecognized technique, despite evidence demonstrating its role in preserving urethral vascularization, a critical determinant of optimal healing. This consideration is particularly important given that urethral necrosis, although rare, may lead to severe complications, including fistula formation, stricture recurrence, or even complete urethral loss.

To facilitate accurate proximal delineation of fibrotic tissue during stricture resection, we describe a modified Hosseini technique, which appears to be a reliable and safe approach. In contrast to the original description, which utilized a fine needle, a gray venous catheter is introduced, allowing the passage of a hydrophilic guidewire. Progressive dilation over the guidewire enables controlled and safe resection under direct vision until healthy proximal urethral tissue is clearly identified.

While sparing the bulbar arteries has been demonstrated to be a rational approach whenever feasible, its combination with our modified Hosseini technique appears, based on our experience, to be feasible, reproducible, and conducive to a faster and more straightforward surgical execution.

## Editorial

In modern reconstructive urology, surgeons aim to optimize outcomes by preserving critical anatomical structures such as muscles, nerves, and vessels, with the dual goals of maintaining sexual function and minimizing secondary complications. Techniques such as one-sided dissection, non-transecting urethroplasty, and bulbar artery-sparing approaches have been introduced to maintain better urethral vascularity.

The primary aim of this article is to highlight the limited adoption of the bulbar artery-sparing technique during posterior post-traumatic urethroplasty, also known as the Gómez technique, despite its strong physiological rationale and well-documented outcomes in the literature. We present our technical modifications, which offer a novel perspective on fibrosis resection to simplify the procedure and facilitate faster surgical execution, based on expert opinion and cumulative surgical experience.

The initial rationale for developing the bulbar artery-sparing technique was to facilitate safer sphincter cuff placement in patients with bulbar strictures following radical prostatectomy. Jordan first described this approach [1], proposing that preservation of the proximal corpus spongiosum blood supply could decrease the risk of cuff-related atrophy and erosion in patients who might subsequently require artificial urinary sphincter implantation.

Sparing of the bulbar arteries during posterior post-traumatic urethroplasty is a relatively little-known technique, first described by Gomez in 2015 [2]. In his most recent large series [3], he demonstrated both the feasibility and the advantages of this modification with long-term follow-up, which clearly preserves the urethral vascular supply-a factor that should be regarded as a true priority in all urethral surgeries.

Joshi et al. have reported the largest published series on urethral necrosis following anastomotic urethroplasty, highlighting the potential severity of this complication [4]. Urethral necrosis, although rare, can have devastating consequences, including stricture recurrence, fistula formation, and even complete loss of urethral function. Given that the vascular supply plays a central role in urethral healing and tissue viability, preserving the bulbar arteries during anastomotic urethroplasty may provide a significant protective advantage. Incorporating a bulbar artery-sparing approach not only enhances urethral vascularity but may also substantially reduce the risk of ischemic complications, thereby improving long-term surgical outcomes.

In our view, there is no rational basis for detaching the corpus spongiosum from the perineum, as this maneuver does not contribute any meaningful increase in flexibility or length to the distal urethra. On the contrary, such unnecessary dissection may compromise the surrounding vascular and neural support, potentially increasing the risk of ischemia, fibrosis, or functional impairment. Preservation of the natural attachments of the spongiosum, therefore, appears to be both anatomically and physiologically sound, ensuring better tissue viability and minimizing avoidable complications.

Morocco is a developing country that is still progressing in terms of healthcare infrastructure and road safety systems. Road traffic accidents remain a major public health problem and represent the leading cause of pelvic fractures, which in turn are strongly associated with posterior urethral injuries. In 2024, Morocco recorded 3,641 fatalities from traffic accidents. There were over 142,000 traffic accidents in 2024, a 15.2% increase compared to 2023 (Table 1).

**Table 1:** Technical fact sheet for the year 2024, according to the official website of the Moroccan agency of road safety

	Road accidents	Killed	Severely injured	Slightly injured
Annual report 2024	142.247	3.641	9.334	192.854
Change 2024/2023	+ 15,2%	+ 4,4%	+ 8,6%	+ 14,9%

Although there are no official national statistics specifically addressing the incidence of pelvic fracture-related urethral injuries in Morocco, clinical experience suggests that the burden is considerable. The frequency appears higher compared with neighboring developed countries, likely due to a combination of factors, including increased rates of road traffic accidents, insufficient trauma systems, and limited access to immediate specialized care.

In our experience, all patients with pelvic fracture urethral injury (PFUI) were treated using the Gómez technique. A total of 34 patients were managed between 2019 and 2024 across two tertiary hospitals. Although Gómez's technique was described as efficient by numerous studies, the objective of the present work is to share our real-world experience, confirming the feasibility and reproducibility of the Gómez technique, while highlighting our technical modifications aimed at simplifying and accelerating the procedure. Our unpublished results demonstrate functional outcomes comparable to those reported by Gómez.

In our experience, the procedure is relatively challenging and consists of a meticulous dorsal dissection of the bulbar and membranous urethra without cutting the bulbar part of the urethra. Once adequate exposure is achieved, the proximal bulbar urethra or the membranous urethra is incised longitudinally along its dorsal aspect, at the most proximal point accessible to the bougie introduced through the external meatus.

**Figure 1:**
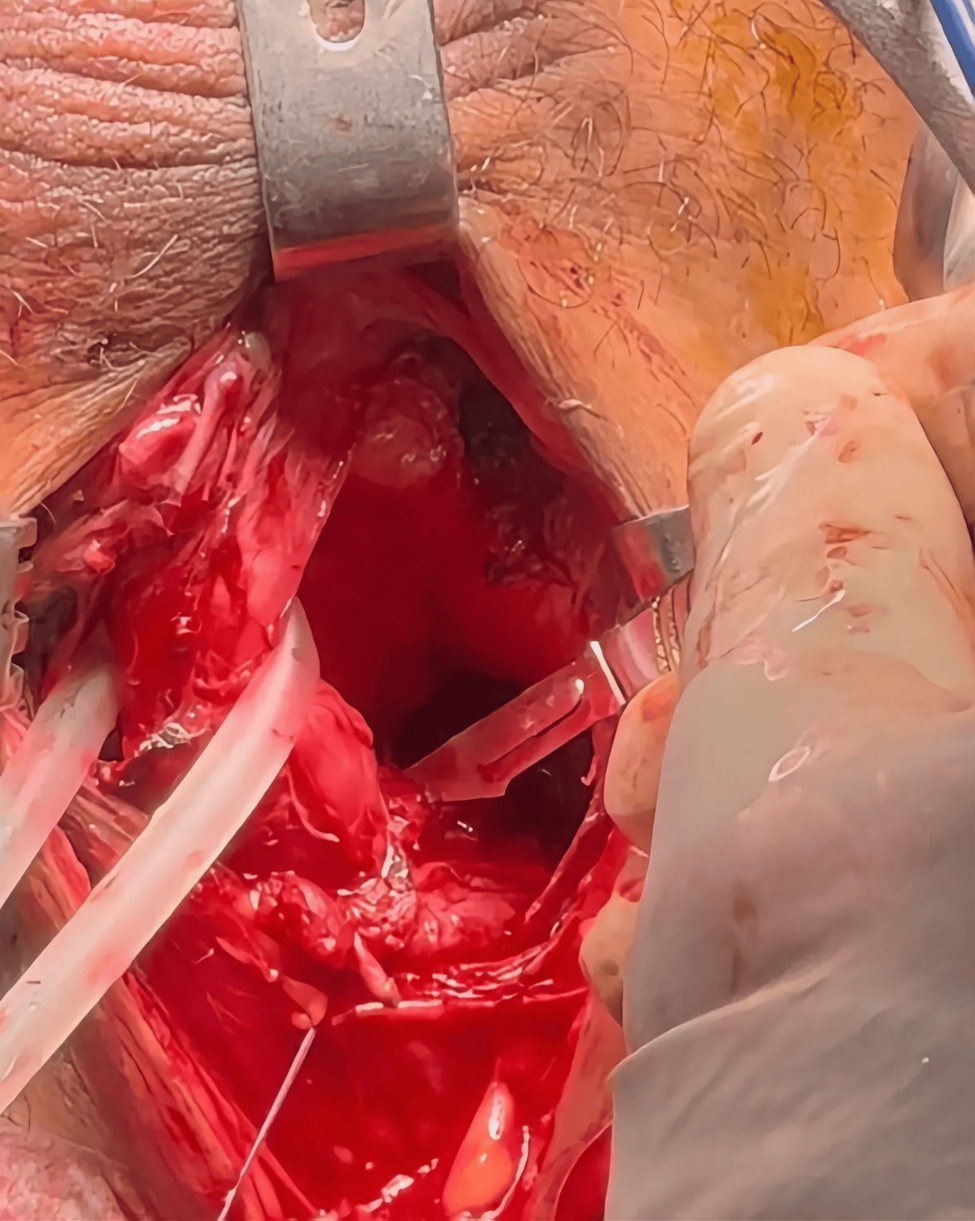
Preserving bulbar arteries during anastomotic urethroplasty Dissection is carried out along the dorsal side of the membranous urethra.

Once the urethra is opened longitudinally, the ventral aspect of the membranous urethra is clearly delineated, which allows accurate identification of the ventral extension of fibrosis. Resection of the fibrous callus is then carried out with care, avoiding deeper ventral dissection to protect the bulbar arteries from inadvertent injury. Unlike Gomez, we do not routinely use Doppler ultrasound to localize the exact course of the bulbo-urethral arteries. Instead, we rely on meticulous dorsal dissection combined with opening the distal healthy urethra, which provides a reliable anatomical landmark and naturally defines the safe limit of ventral dissection, thereby minimizing the risk of compromising the arterial supply during fibrosis resection (Figure 1).

The proximal delimitation of fibrosis resection can sometimes be challenging. This step is facilitated using the Hosseini technique, which we have systematically incorporated into our practice. The original Hosseini technique [5] employs antegrade flexible cystoscopy through the suprapubic catheter tract to guide the insertion of a fine needle into the proximal end of the urethra.

**Figure 2:**
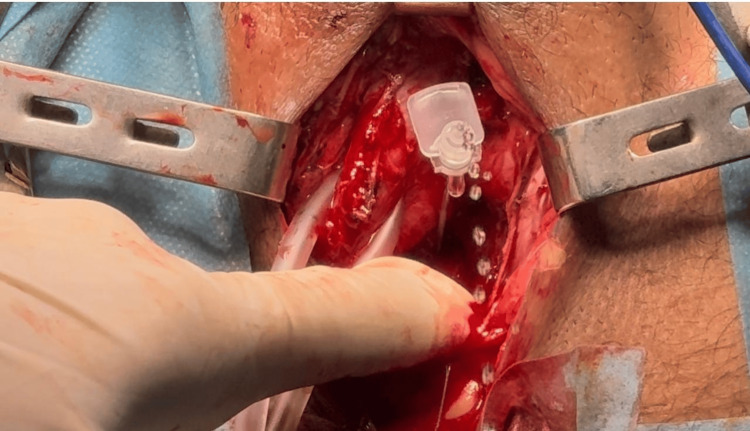
Modified Hosseini technique Outflow of urine confirming entry of a gray venous catheter into the urethral lumen.

**Figure 3:**
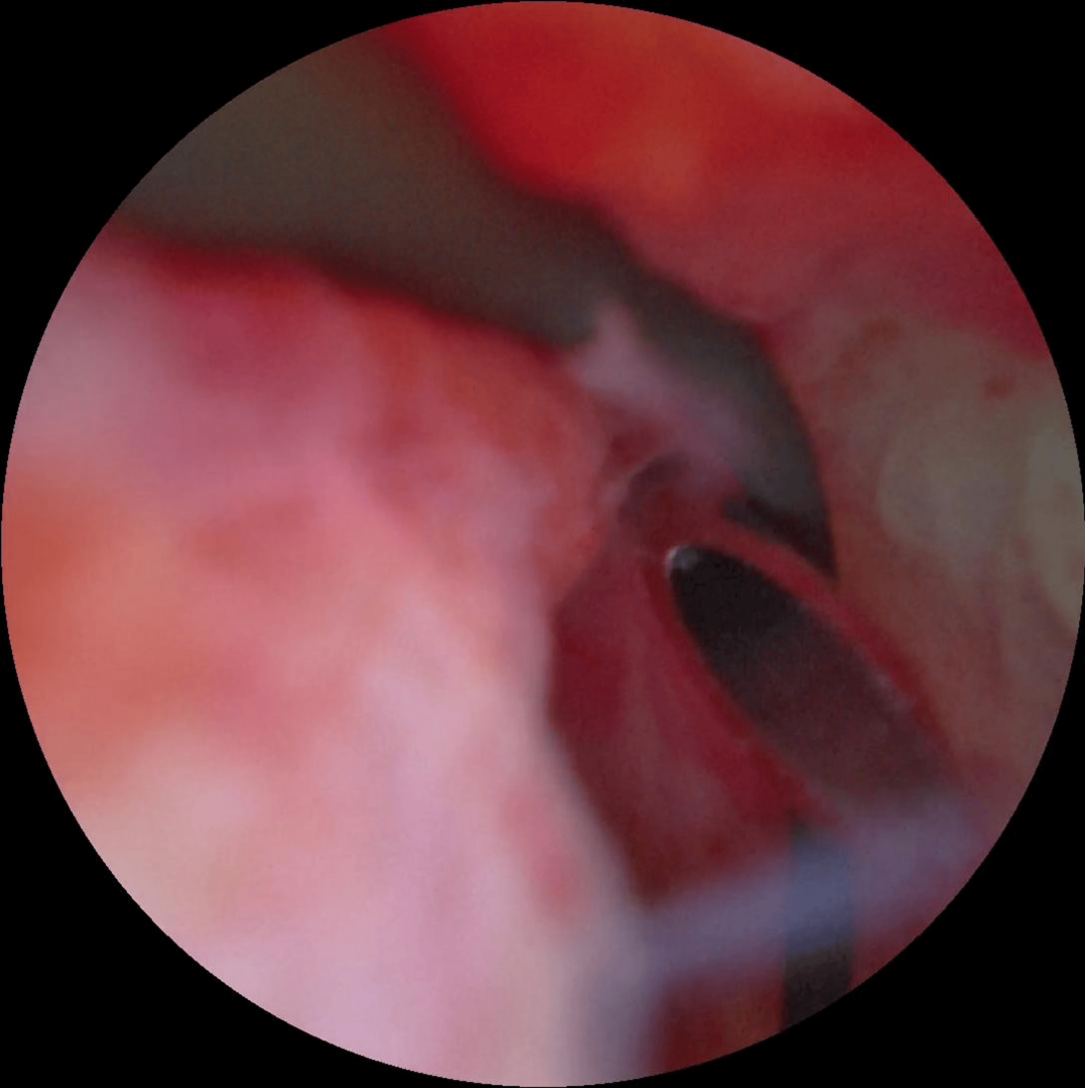
Modified hosseini technique The tip of the needle is oriented toward the flexible cystoscope within the proximal urethra.

**Figure 4:**
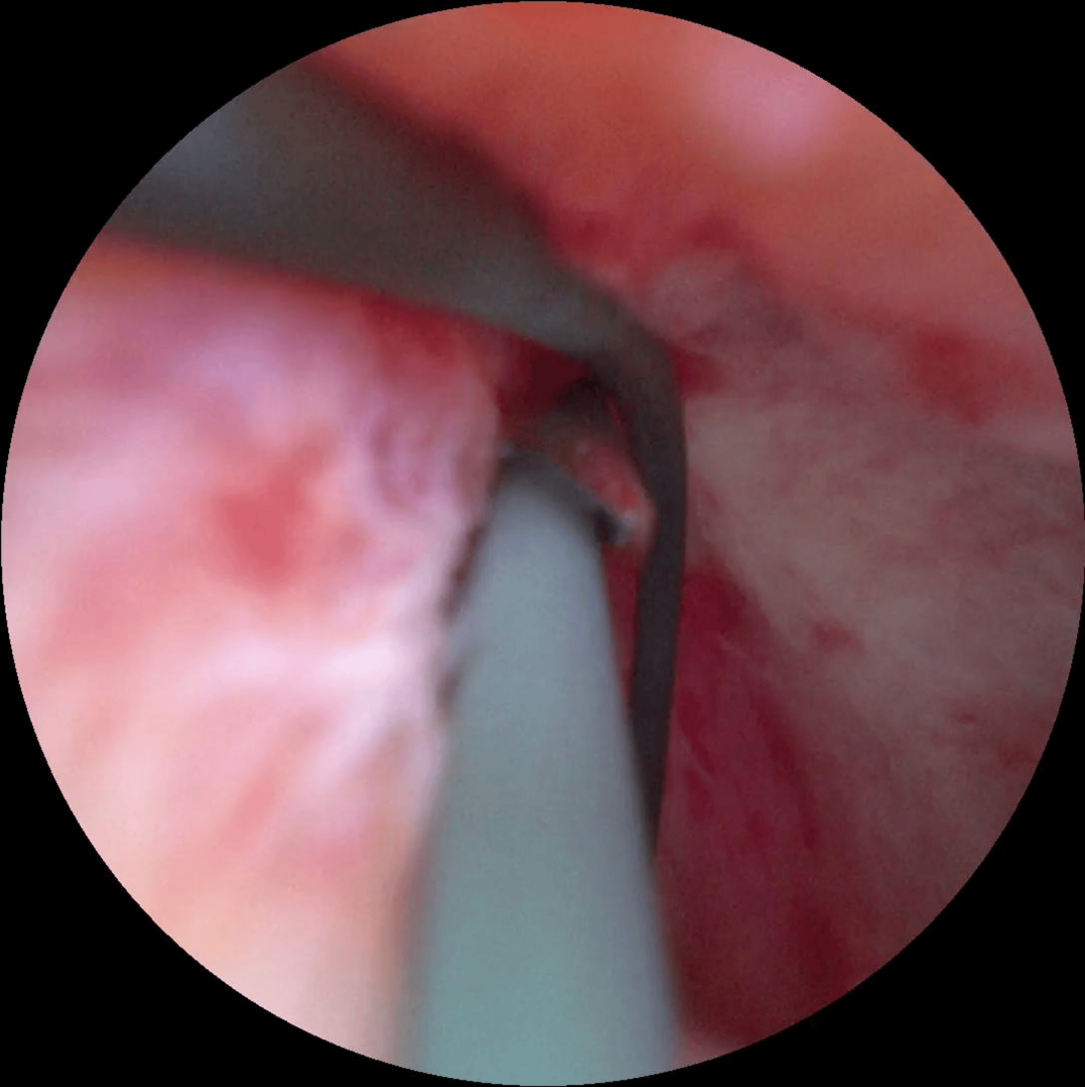
Modified Hosseini technique Introduction of the guidewire through the needle to allow subsequent dilation.

In our practice, we use a modified version of this technique: instead of a fine needle, we introduce a gray venous catheter, which allows subsequent placement of a hydrophilic guidewire (Figures 2, 3, 4).

Dilatation of the guidewire’s trajectory through the scarry fibrotic segment (Video 1) enables controlled subsequent resection under direct visualization, progressing safely until reaching proximal healthy urethral tissue (Video 2).

**Video 1:** Modified Hosseini technique Dilatation through the fibrotic scar allows oriented and limited scar resection.

**Video 2:** Modified Hosseini technique Resection of the fibrotic tissue.

One of the key points deduced from Gomez’s article is that vessel preservation does not hinder approximation of the urethral ends, which can still be achieved using Webster’s progressive approach. Spatulation is performed on the dorsal aspect of the distal urethra, where the corpus spongiosum is less developed, and the anastomosis is then completed with six interrupted sutures. The combination of these techniques offers a straightforward and effective method to achieve a tension-free urethral anastomosis while maintaining optimal vascular integrity.

Key technical steps

Bulbar Artery Preservation

Whenever possible, preserve the bulbar arteries by favoring a dorsal dissection approach until the fibrotic obstructed segment is reached.

Intraoperative Antegrade Cystoscopy

Perform antegrade cystoscopy during the procedure to accurately delineate the proximal limit of the urethral stricture.

Access Through the Fibrotic Segment

Insert a gray venous catheter through the fibrotic area, aiming toward the "culmen" point of the proximal urethral lumen.

Guidewire Placement

Advance a hydrophilic guidewire through the needle into the proximal urethra.

Needle Removal

Carefully withdraw the needle while maintaining the guidewire in place.

Progressive Dilation

Introduce sequential soft plastic dilators over the guidewire to gradually dilate and achieve controlled opening of the fibrotic urethral segment. Avoid metallic dilatators when sharp angulation is suspected, as they risk perforation or trauma due to their rigidity.

Targeted Fibrosis Resection

Perform controlled and progressive resection of the fibrotic tissue with small tissue fragments until healthy urethral tissue is reached, controlled by antegrade cystoscopic evaluation.

Urethral Approximation Assessment

Perform an urethral approximation test and apply Webster’s progressive mobilization steps if a tension-free anastomosis is not initially achievable.

Anastomosis and Catheterization

Complete the urethral anastomosis using six evenly distributed sutures, ensuring mucosa-to-mucosa alignment, followed by insertion of a 14 Fr bladder catheter advanced preferentially over the guidewire.
